# Molecular imaging HDACs class IIa expression-activity and pharmacologic inhibition in intracerebral glioma models in rats using PET/CT/(MRI) with [^18^F]TFAHA

**DOI:** 10.1038/s41598-019-40054-2

**Published:** 2019-03-05

**Authors:** Maxwell T. Laws, Robin E. Bonomi, Swatabdi Kamal, David J. Gelovani, Jeremy Llaniguez, Shreya Potukutchi, Xin Lu, Thomas Mangner, Juri G. Gelovani

**Affiliations:** 1 Department of Biomedical Engineering, College of Engineering and School of Medicine, Wayne State University, Detroit, MI 48202 USA; 2 Positron Emission Tomography Center, Wayne State University, Detroit, MI 48202 USA; 3 Department of Oncology, Wayne State University, Detroit, MI 48202 USA; 4 Department of Neurosurgery, Wayne State University, Detroit, MI 48202 USA; 5 Molecular Imaging Program, Karmanos Cancer Institute, Wayne State University, Detroit, MI 48201 USA

## Abstract

HDAC class IIa enzymes (HDAC4, 5, 7, 9) are important for glioma progression, invasion, responses to TMZ and radiotherapy, and prognosis. In this study, we demonstrated the efficacy of PET/CT/(MRI) with [^18^F]TFAHA for non-invasive and quantitative imaging of HDAC class IIa expression-activity in intracerebral 9L and U87-MG gliomas in rats. Increased accumulation of [^18^F]TFAHA in 9L and U87-MG tumors was observed at 20 min post radiotracer administration with SUV of 1.45 ± 0.05 and 1.08 ± 0.05, respectively, and tumor-to-cortex SUV ratios of 1.74 ± 0.07 and 1.44 ± 0.03, respectively. [^18^F]TFAHA accumulation was also observed in normal brain structures known to overexpress HDACs class IIa: *hippocampus*, *n*.*accumbens*, PAG, and cerebellum. These results were confirmed by immunohistochemical staining of brain tissue sections revealing the upregulation of HDACs 4, 5, and 9, and HIF-1α, hypoacetylation of H2AK5ac, H2BK5ac, H3K9ac, H4K8ac, and downregulation of KLF4. Significant reduction in [^18^F]TFAHA accumulation in 9L tumors was observed after administration of HDACs class IIa specific inhibitor MC1568, but not the SIRT1 specific inhibitor EX-527. Thus, PET/CT/(MRI) with [^18^F]TFAHA can facilitate studies to elucidate the roles of HDAC class IIa enzymes in gliomagenesis and progression and to optimize therapeutic doses of novel HDACs class IIa inhibitors in gliomas.

## Introduction

Over the past two decades, several studies have demonstrated that GBM progression and recurrence is linked to epigenetic mechanisms including, mutations in IDH1/IDH2 genes, epigenetic modifying enzymes, histone deacetylases (HDACs), histone methyltransferases (HMTs), DNA methyltransferases (DNMT) and various DNA demethylases^1^. Many novel inhibitors of these key epigenetic regulatory enzymes have been developed and tested in clinical trials^2^. It has been demonstrated, that HDAC inhibitors sensitize gliomas and other types of cancer to chemotherapeutic agents and radiation, including: trichostatin (TSA), SAHA (vorinostat), valproic acid (VPA), LBH589 (panobinostat), MS275 (entinostat), PXD101 (belinostat)^3–19^. Studies in patient-derived glioblastoma cells demonstrated that SAHA, VPA, MS275, LBH589 and Scriptaid are effective radiosensitizers of gliomas^20^. In particular, panobinostat (LBH589) increased the cytotoxic effect of radiation therapy plus TMZ in U251 cells with low MGMT expression more significantly than in T98G cells, expressing MGMT at high levels^21^. In contrast, SAHA potentiated the evolution of acquired TMZ resistance, which was linked to upregulation of MGMT expression in patient-derived GBM xenografts^4^. Short-term and long-term treatments with valproic acid modulated DNA methylation and differentiation behavior, but not temozolomide sensitivity^19^. Although, the vast majority of ongoing clinical trials in GBM use drugs that predominantly inhibit HDAC class I enzymes (valproic acid, vorinostat, belinostat, entinostat, panobiostat)^6,22–24^, pharmacologic inhibition of HDAC class I enzymes may not be the most effective approach to potentiate TMZ and radiation therapy of GBM.

A growing number of reports underscore the important roles of HDAC class IIa enzymes (HDAC4, 5, 7, 9) in GBM progression^3,25–29^, invasion^30–32^, responses to TMZ and radiotherapy^4,21,22^, and prognosis^33^. However, no studies have been reported to date on the efficacy of HDACs class IIa-selective inhibitors alone or in combination with TMZ and radiation therapy of GBM. Previous studies in non-CNS tumors, aimed to identify targets for radiosensitization using siRNA and shRNA-mediated knockdown of individual HDAC isoforms, revealed HDACs class IIa as potential targets for pharmacologic inhibition^12,34–38^. Recently, it was demonstrated that that the level of HDAC4 expression of GBMs negatively correlates with overall survival rates after temozolomide and radiation therapy^39,40^. Moreover, high HDAC4 expression was found to be a strong predictive factor of poor outcome of TMZ treatment of GBM, independent of MGMT status and Ki67 index. *In vitro* experiments revealed that shRNA-mediated silencing of HDAC4 radiosensitized the U87MG and U251MG cells by promoting the accumulation of DNA double-strand breaks (DSBs), by affecting the molecular machinery of DSBs repair, and by promoting the radiation therapy-induced senescence. In contrast, the upregulation of HDAC4 expression sustained GBM stem-like radiation-resistant phenotype^40^. Also, it was demonstrated that p53(wt) is a key downstream target of the GBM radiosensitization induced by silencing of HDAC4, because the overexpression of p53(mt) active isoform resulted in restrain of radiosensitization by targeting HDAC4^40^.

Controversially, other studies reported opposite results; namely, that patients with methylated MGMT promoter and higher expression of HDAC4 had better survival after TMZ and radiation therapy^33,41^. Altogether, these observations suggest that HDACs class IIa and, in particular, the HDAC4 play key roles in determining responses to radiation-induced DNA damage and in maintaining cellular “stemness”, thus promoting radioresistance. HDACs class IIa represent both, prognostic biomarkers and potential therapeutic targets in GBM. Therefore, non-invasive molecular imaging of expression-activity of HDAC class IIa enzymes may help in identification of GBM patients who may benefit from the addition of HDAC class IIa inhibitors to conventional TMZ-radiotherapy to improve the survival and overall outcome.

Previously, we developed 6-(tri-fluoroacetamido)-1-hexanoicanilide ([^18^F]TFAHA), a highly-selective radiotracer for quantitative imaging of HDAC class IIa enzyme expression-activity *in vivo* using PET/CT/(MRI)^42^. Current studies demonstrated efficacy of PET/CT/(MRI) with [^18^F]TFAHA for imaging HDACs class IIa expression-activity in 9L and U87-MG brain glioma models in rats, and for non-invasive monitoring of MC1568 induced inhibition of HDAC class IIa activity in 9L gliomas. Thus, non-invasive repetitive PET/CT/(MRI) with [^18^F]TFAHA may facilitate future clinical studies aimed to elucidate the roles of HDAC class IIa enzymes in gliomagenesis and progression and to optimize therapeutic doses of novel HDACs class IIa inhibitors in combined chemo-radiotherapy of GBM.

## Results

### [^18^F]TFAHA PET/CT/(MRI) of HDACs class IIa expression-activity in intracerebral 9L gliomas in rats

**Figure 1:**
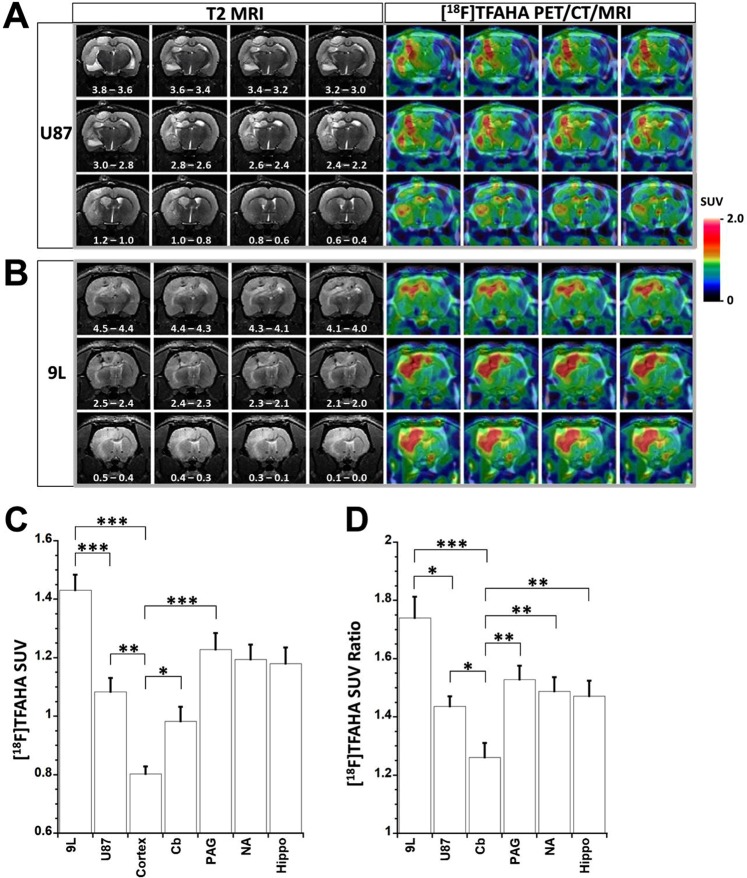
PET/CT/(MRI) of HDACs class IIa expression in intracerebral glioma models in rats. Representative series of coronal images of the rat brain bearing intracerebral U87-MG glioma (**A**) and 9L gliosarcoma (**B**). The position of sections relative to *bregma* is indicated in mm on T2-weighted MR images. [^18^F]TFAHA PET/CT images were obtained at 20 minutes post injection of radiotracer and co-registered with T2-weighted MR images. The levels of [^18^F]TFAHA accumulation in tumors and different structures of the brain were measured in SUV (**C**) and SUV ratio normalized by the SUV of the contralateral cortex (**D**) for 9L (N = 10) and U87-MG (N = 9) gliomas. PET/CT images are color-coded to standard uptake values (SUV). Data - mean ± SEM. Statistical significance was determined via one-way ANOVA, *denotes p < 0.05, **denotes p < 0.01, ***denotes p < 0.001.

PET/CT(MRI) with [^18^F]TFAHA demonstrated heterogeneously increased, transient accumulation of [^18^F]TFAHA-derived radioactivity in i.c. 9L (Fig. 1A; N = 10) and U87-MG (Fig. 1B; N = 9) tumors. The maximum contrast between tumors, versus white matter and cortex was observed at 20 min post i.v. administration of [^18^F]TFAHA, resulting in SUV of 1.45 ± 0.05 for 9L and 1.08 ± 0.05 for U87-MG gliomas (Fig. 1C) and tumor-to-cortex SUV ratios of 1.74 ± 0.07 for 9L and 1.44 ± 0.03 for U87-MG gliomas, respectively (Fig. 1D). Also, increased levels of retention of [^18^F]TFAHA-derived radioactivity were observed in normal structures of the brain that are known to express higher levels of HDACs class IIa, including: *hippocampus*, *n*.*accumbens*, PAG, and cerebellum (Fig. 1**)**. Similar magnitude of [^18^F]TFAHA time-activity curves (TACs) were observed during the first few minutes after intravenous administration in normal brain structures expressing high levels of HDACs class IIa (i.e., n.accumbens, PAG, cerebellum), as the initial magnitude of TACs observed in 9L gliomas (Fig. S1).

### IHC analyses of brain tissue sections

**Figure 2:**
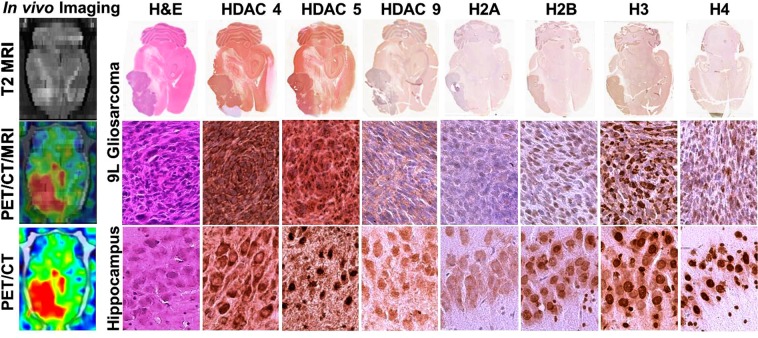
Comparative analysis of [^18^F]TFAHA PET/CT/(MRI) and immunohistochemical (IHC) staining in 9L glioma and in the contralateral *hippocampus*. The left column: (top) axial T2-weighted MR image; (middle) corresponding axial PET/CT/(MRI) fusion image; (bottom) corresponding axial PET/CT image. Macroscopic (top row) and microscopic images (x200) of axial brain sections stained with H&E and by IHC to visualize the levels of expression of HDACs 4, 5, 9, and acetylation of histones H2AK5ac, H2BK5ac, H3K9ac, and H4K8ac in 9L tumor (middle row) and in contralateral hippocampal CA2 (bottom row). PET/CT images are color-coded to standard uptake values (SUV).

**Figure 3:**
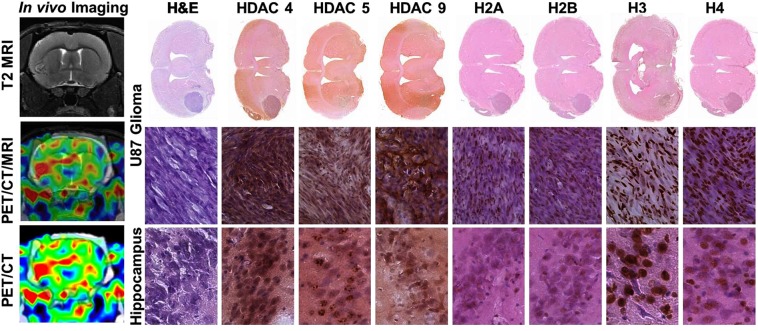
Comparative analysis of [^18^F]TFAHA PET/CT/(MRI) and immunohistochemical (IHC) staining in U87-MG glioma and in the contralateral *hippocampus*. The left column: (top) coronal T2-weighted MR image; (middle) corresponding coronal PET/CT/(MRI) fusion image; (bottom) corresponding coronal PET/CT image. Macroscopic (top row) and microscopic images (x200) of axial brain sections stained with H&E and by IHC to visualize the levels of expression of HDACs 4, 5, 9, and acetylation of histones H2AK5ac, H2BK5ac, H3K9ac, and H4K8ac in U87-MG tumor (middle row) and in contralateral hippocampal CA2 (bottom row). PET/CT images are color-coded to standard uptake values (SUV).

**Figure 4:**
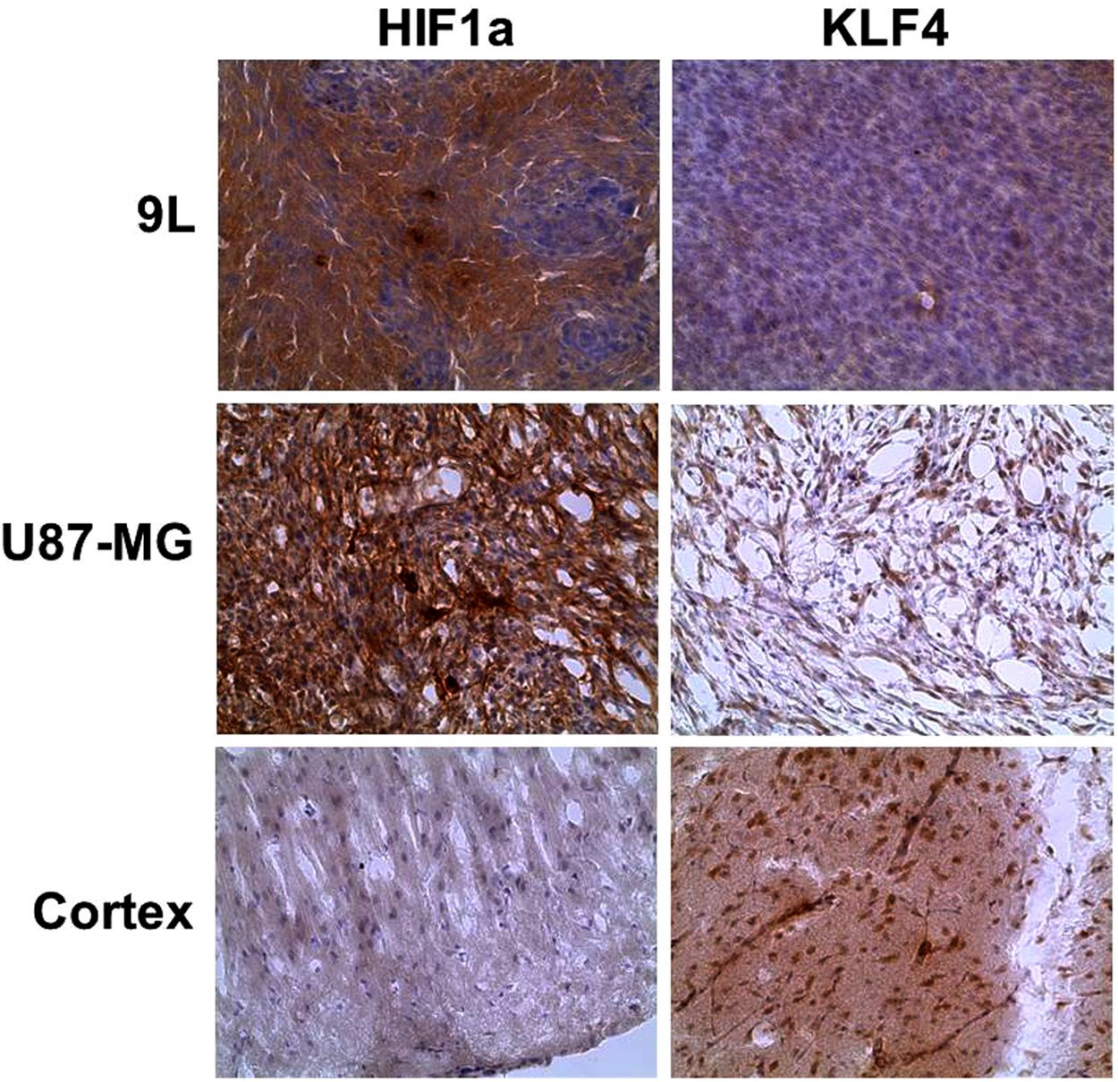
Immunohistochemical staining (brown) for HIF-1α and KLF4 in 9L, U87 gliomas, and in contralateral cortex; counterstaining with hematoxylin (blue).

To validate the results of non-invasive PET/CT/(MRI) of HDACs class IIa expression and to determine which particular isoform has contributed to [^18^F]TFAHA uptake in tumors, 6 animals were sacrificed after the imaging session (N = 3 for each tumor type), their brains extracted for histologic analyses. H&E staining of brain tissue sections confirmed the localization of tumors observed on MRI and PET images. IHC staining for HDACs 4, 5, and 9 demonstrated that the HDACs 4 and 5 are overexpressed in 9L gliomas, as compared to HDACs 9 (Fig. 2). The subcellular localization of HDAC4 was mostly perinuclear with less than 10% cells having nuclear localization. In contrast, HDAC5 had mostly nuclear localization in about 70% cells, although it was also present in the cytoplasm. The level of expression of HDACs 4 and 5 in 9L gliomas was comparable to that in the contralateral hippocampal CA2 and CA3. HDACs 9 showed only faint and mostly cytoplasmic localization in 9L gliomas, which was much lower than in contralateral *hippocampus*. The upregulation of expression-activity of HDACs 4 and 5 in 9L gliomas was co-localizing with hypo-acetylation of histones H2A, H2B, and H4, as compared to contralateral *hippocampus*. Similar patterns of HDAC4, 5, and 9 expression and hypoacetylation of histones were observed in i.c. U87-MG tumors (Fig. 3). Expression of HDACs class IIa was elevated in hypoxic tumor regions expressing high levels of HIF-1α. The U87-MG gliomas expressed HIF-1α at higher levels and more uniformly than the 9L gliosarcomas (Fig. 4). Expression of KLF4 was almost non-detectable in 9L tumors, both in endothelial cells of tumor capilaries and in tumor cells, while U87-MG gliomas expressed low levels of KLF4 in tumor cells. In contrast, high levels of KLF4 expression were observed in endothelial cells of cortical capillaries and in cortical neurons (Fig. 4).

### PET/CT/(MRI) with [^18^F]TFAHA for monitoring the pharmacologic inhibition of HDACs class IIa expression-activity in intracerebral 9L gliomas in rats

**Figure 5:**
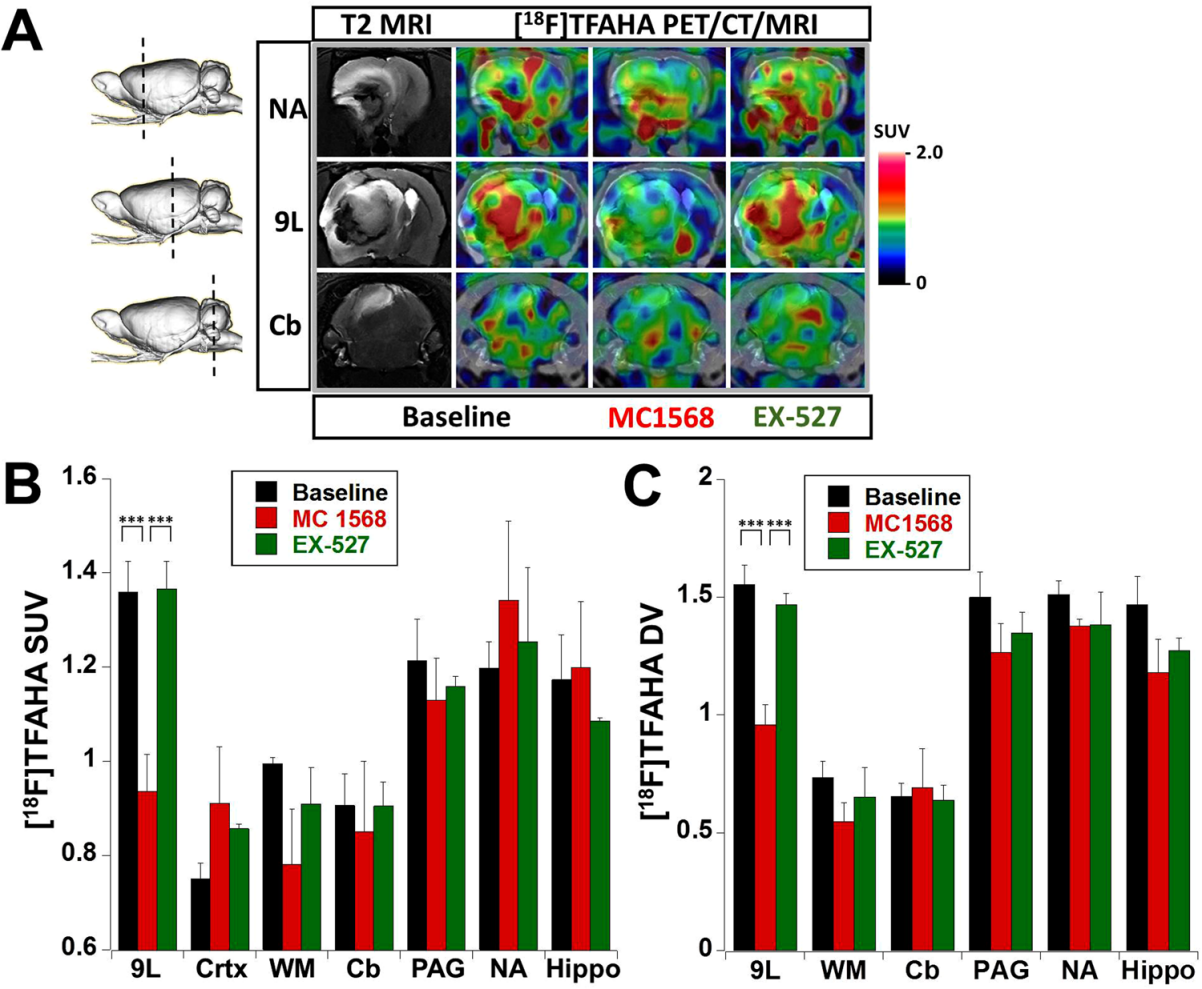
Monitoring pharmacologic inhibition of HDACs class IIa with [^18^F]TFAHA PET/CT/(MRI). (**A**) Representative coronal T2-weighted MRI and fused with [^18^F]TFAHA PET/CT/(MRI) images depicting different regions of the rat brain with intracerebral 9L tumor (shown by a dotted line on a side-view of a 3D rendered image): through the area of *n*.*accumbens* (NA), in the middle of 9L tumor (9L), and through the cerebellum (Cb). [^18^F]TFAHA PET/CT/(MRI) were obtained at 20 min post radiotracer administration before (baseline) and after treatment with either MC1568 (HDAC class IIa- selective inhibitor) or EX-527 (SIRT1-selective inhibitor). PET/CT images are color-coded to standard uptake values (SUV). The levels [^18^F]TFAHA-derived radioactivity are expressed as (**B**) standard uptake values (SUV) or (**C**) distribution volumes (DV) in 9L tumors and different brain structures at baseline (N = 4) and after therapy with MC1568 (N = 3) or EX-527 (N = 3). Data – mean ± SEM. Statistical significance was determined using paired t-test; ***indicates p < 0.005.

Treatment with a single dose of MC1568 (30 mg/kg, i.p., 30 min prior to injection of [^18^F]TFAHA) resulted in the inhibition of HDACs class IIa activity in 9L tumors, as evidenced by statistically significant decreases in SUV (p < 0.005) and DV (p < 0.005) of [^18^F]TFAHA (Figs 5 and S1–5**)**. Although a similar trend for reduced SUV and DV of [^18^F]TFAHA after treatment with MC1568 was observed in the contralateral white matter, *hippocampus*, *n*.*accumbens*, periaqueductal gray matter (PAG), it was not statistically significant. Treatment of rats with a single dose of SIRT1 inhibitor EX-527 (5 mg/kg, i.p., 30 min prior to injection of [^18^F]TFAHA) did not inhibit the HDACs class IIa activity neither in 9L tumors, nor in the contralateral brain structures, PAG and cerebellum, as evidenced by the lack of statistically significant decreases in [^18^F]TFAHA SUV and DV, as compared to baseline levels (Figs 5 and S1–5).

## Discussion

In the current study, we demonstrated that PET/CT/(MRI) with [^18^F]TFAHA allows a non-invasive, quantitive visualization of HDAC class IIa expression-activity in U87-MG and 9L intracerebral glioma models in rats. We have chosen these two glioma models in rats because of their vastly different pathomorphological and intracerebral growth charcteristics The 9L rat is a gliosarcoma grows in the rat brain with prolific angiogenesis, well definable tumor margins, and minimal inavsion into the peritumoral and/or distal brain regions^43^. In contrast, the U87-MG human glioblastoma is characterized by less active angiogenesis and a more diffuse, infiltrative growth pattern in the brain of immune compromized rats^44^. Expression of HDACs 4 and 5 and consequences of their inhibition or knockdown have been previously investigated in U87-MG cell line^26,28,45^. It has been previously established that HDAC4 interacts with Sp1 and reduces acetylation of histones H3 and H4 in the proximal promoter region of p21^WAF1/Cip1^, which causes down-regulation of p21(WAF1/Cip1) expression^46^. Silencing of HDAC4 reactivates p21(WAF1/Cip1) and causes cancer cell growth arrest *in vitro* and inhibits tumor growth in an *in vivo* U87-MG glioma model^26^. HDAC5 is constitutively upregulated in U87MG, and in U251MG, T98G, and LN-229 glioma cell lines; HDAC5 promotes their proliferation by upregulation of Notch 1, whereas the knockdown of HDAC5 by siRNA suppressed proliferation and induced apoptosis in these glioma cell lines^28,45^.

Consistent with [^18^F]TFAHA accumulation in 9L and U87-MG gliomas, higher levels of expression of HDACs class IIa (specifically HDACs 4, 5, and 9) were confirmed by IHC analyses of brain tissue sections, obtained after completion of non-invasive PET/CT/(MRI). The levels of HDACs 4,5, and 9 expresison in these glioma models were comparable to normal structures of the brain, known to express higher levels of these enzymes, including *n*.*accumbens*, *hippocampus*, PAG, and cerebellum^42,47^. Also, the observed upregulation of HDACs class IIa expression-activity in 9L and U87-MG tumors was associated with hypoacetylation of histones H2A, H2B and H4. This observation is consistent with previous reports of HDAC4/N-CoR/HDAC3 complex binding via HDAC4 to DNA-bound different transcription factors, which allows HDAC3 to deacetylate histones and to repress basal transcription^48^. However, the reason for the observed hyper-acetylation of H3K9 in presence of upregulated expression-activity of HDACs class IIa remains unclear and warrant additional studies.

The magnitude of HDACs class IIa in 9L and U87-MG tumors was heterogeneous, as evidenced by non-uniform distribution of [^18^F]TFAHA within the MRI-detectable tumors. This is partially due to higher levels of HDAC4 expression in hypoxic tumor regions with increased levels of HIF1α expression, as demonstrated using IHC. These results are consistent with previously published data demonstrating that members of HDACs class IIa, specifically HDAC4 and HDAC5, are phosphorylated by AMPK in response to hypoxia and nutrient insufficiency, which promotes their nuclear export^49^. In the cytosol, HDAC4 and HDAC5 stabilize HIF-1α by deacetylating Hsp70, promoting the transfer of HIF-1α from Hsp70 to Hsp90 for the completion of the maturation process. Therefore, while the AMPK may reduce the translation of HIF-1α, phosphorylated HDACs 4 and 5 compensate it by enhancing the efficiency of posttranslational Hsp-facilitated folding of HIF-1α, thus ensuring HIF-1 activation under stressful conditions^50^. Also during such stress, HIF-1α accumulates in the nuclei where PCAF acetylates its N-terminal lysines between AA11-21 and at position 674 in its inhibitory domain. Structural zinc-binding domain of HDACs class IIa is crucial for their functions^31^, including HIF-1 transcriptional complex formation and function^51–56^. HDAC4/N-CoR/HDAC3 complexes bind via HDAC4 to the acK674 of HIF-1 and causing HDAC3-mediated deacetylation of N-terminal lysines between AA11-21 of HIF-1. Deacetylation of HIF-1 N-terminal lysines located between AA11-21 facilitates dissociation of HIF-1α from FIH-1 and binding with p300HAT and HIF-1β needed for formation of HIF-1 complex, which via p300 acetylates histones in cell survival/angiogenesis genes, thus stimulating their transcription^53,55^. The knockdown of either HDAC4 or HDAC5 reduces HIF-1α protein levels, suppresses HIF-1 functions^57^ and downregulates VEGF expression^53^, reduces the HIF-1α-mediated increase of glycolysis and resistance to docetaxel in Hep3B cells^54^.

Also, it has been demonstrated that MEF2, which is the major partner protein forming functional complexes with HDAC4 (class IIa), is a transcriptional target of HIF1α^58^. The MEF2D is overexpressed in gliomas, and in complex with HDAC4 (MEF2D/HDAC4) promotes the tumorigenicity of glioma cells (i.e., U87-MG, U251-MG)^59^. MEF2 is a converging hub for HDAC4 and PI3K/Akt-induced malignant transformation^60^. MEF2 transcriptionally upregulates the expression of Krüppel-like factor 4 (KLF4) transcription factor, which in turn transcriptionally upregulates the expression of zonula occluden-1, claudin-5, and occludin, that are important structural components of blood brain barrier capillaries. Overexpression of miR-18a negatively regulates MEF2D and increases the permeability of the blood-tumor barrier via KLF4-mediated downregulation of zonula occluden-1, claudin-5, and occludin^61^. These reports may explain our current observations of the lack of KLF4 expression in 9L and U87-MG gliomas with increased HDAC4 and HIF1α expression. It is conceivable, that in gliomas high levels of HDAC4 expression in complex with MEF2 acts as transcriptional repressor of KLF4 and, consequently, of zonula occluden-1, claudin-5, and occludin gene expression, which may be another important component in the mechanism of BBB disruption in gliomas. The HDAC4/MEF2-mediated suppression of BBB structural integrity provides the rationale for therapy of GBM with HDAC4-specific inhibitors, that may un-repress the expression of BBB-specific proteins, decrease BBB “leakage”, induce “vascular normalization”, and reverse (or bypass) the mechanisms of resistance to VEGFR inhibitors (i.e., bevacizumab). Because HDAC4 positively regulates HIF-1α stability and transcriptional activity and negatively regulates transcriptional activity of MEF2^62–64^, pharmacologic inhibition of HDAC4 with HDACs class IIa selective inhibitors represents a promising approach to therapy of GBM and may have synergistic effects when combined with radiotherapy.

The MC1568 is a selective inhibitor of class II HDACs activity^65–67^, which promotes nuclear import and proteasome-mediated degradation of HDAC4 and HDAC5 in the nucleus of the cell^68^. Previously, MC1568 has been used to inhibit HDACs 4 and 5 in pre-clinical studies of the mechanisms of amphetamine and alcohol addiction^69^, neuronal remodeling after stroke^70^, and myogenesis^71^. However, no pre-clinical (or clinical) studies of the efficacy of pharmacologic inhibition of HDACs class IIa in gliomas have been reported to date. Current study demonstrated that MC1568 administered in rats as a single i.p. dose of 30 mg/kg, 30 min prior to administration of [^18^F]TFAHA, down-regulates the activity of HDACs class IIa (predominantly HDACs 4 and 5) in intracerebral 9L gliomas. Pretreatment of 9L tumor-bearing rats with MC1568 at this dose did not significantly reduce the magnitude of HDACs class IIa expression-activity in the contralateral brain structures (i.e., *n*.*accumbens*, *hippocampus*) and in cerebellum, that express HDACs 4 and 5 at similar or higher levels as 9L tumor cells (as demonstrated using IHC). Such differences in the magnitude of HDACs class IIa inhibition in 9L gliomas versus contralateral brain structures can be explained, at least in part, by higher concentrations of MC1568 delivered to the 9L tumor tissue through the leaky tumor microvasculature, as compared to relatively lower concentrations of MC1568 in the contralateral brain structures with non-compromised blood-brain-barrier (BBB).

Furthermore, the results of Logan graphical analyses demonstrated that the decrease in transient retention and distribution volume (DV) of [^18^F]TFAHA-derived radioactivity in 9L gliomas after administration of MC1568 results mainly from an acute inhibition of HDACs class IIa activity, whereas no changes in the DV of [^18^F]TFAHA-derived radioactivity were observed in the same 9L tumors after pre-treatment with SIRT1-specific inhibitor EX-527, as compared to baseline. Considering the mechanism of action of MC1568, it is unlikely that pretreatment with MC1568 by i.p. route 30 min prior to injection of [^18^F]TFAHA could have caused an acute reduction (normalization) of BBB permeability in the 9L tumors, that could explain the reduction in the apparent DV of [^18^F]TFAHA-derived radioactivity. Moreover, high lipophilicity of [^18^F]TFAHA (LogP = 2.34) enables it to diffuse across the capillaries with intact BBB and rapidly equilibrate between blood and tissue compartments in the normal brain regions. The latter is evidenced by the high level of [^18^F]TFAHA in the normal brain observed with PET during the first few minutes after intravenous administration and higher SUVs and DVs of [^18^F]TFAHA-derived radioactivity in the brain structures expressing high levels of HDACs class IIa. However, using a single tracer, such as [^18^F]TFAHA, it is hard to determine the degree to which a single dose of MC1568 may influence BBB permeability or perfusion and, thus, the delivery of [^18^F]TFAHA in 9L gliomas. This question could be addressed using multi-tracer approaches or dynamic contrast-enhanced MRI (DCE-MRI).

In the current study only a partial inhibition of HDACs class IIa activity was achieved using a single i.p. dose of 30 mg/kg, 30 min prior to administration of [^18^F]TFAHA, which was probably because the dose of MC1568 was not sufficiently high. Significantly higher cumulative doses and chronic repetitive administration of MC1568 have been used in other studies to inhibit the HDACs class IIa activity in the brain. For example, in one study, MC1568 was administered in 6.5 mg/kg doses i.p. every other day for a 23-day period to assess the effects of HDACs class II inhibition in neonatal mice on glucose levels in blood, oxidative metabolism in skeletal muscle and adipose tissue^72^. In another study, to explore the effect of selective degradation of HDAC4 and HDAC5 on cocaine addiction–like behaviors, 0.5 mg/kg MC1568 was administered i.p. daily for 10 consecutive days demonstrating 35% decrease in HDACs class IIa activity in the *n*.*accumbens* and no inhibition of HDAC class I enzymes^69^. The highest previously reported dose of MC1568 was 40 mg/kg administered to rats 4 times during the first two weeks postnatal^73^. In the current study, the specificity of MC1568 for inhibition of HDACs class IIa in 9L gliomas was demonstrated by the lack of changes in [^18^F]TFAHA accumulation in 9L tumors after pre-treatment of rats with a SIRT1–specific inhibitor EX-527^74^ (5 mg/kg, i.v. 30 min before administration of [^18^F]TFAHA). Also, pre-treatment with EX-527 had no effect on the magnitude of [^18^F]TFAHA accumulation in the contralateral brain structures, known to overexpress HDACs class IIa enzymes under normal conditions (i.e., *n*.*accumbens*, *hippocampus*, cerebellum). The dose of EX-527 for i.v. administration used in this study was within the range of doses reported for single and repetitive i.p. administration (10 mg/kg, i.p. for 4 days) in mice and rats that caused cognitive and behavioral changes^75–77^, indicating that EX-527 crosses BBB upon systemic administration.

Prommising results of the current study warrant further investigations of HDACs class IIa inhibitors in combination with conventional chemo-radiation therapies, in which PET/CT(MRI) with [^18^F]TFAHA of HDAC class IIa expression-activity levels in GBM may serve as a predictive biomarker of treatment response. Predictive biomarkers are vitally important when testing drugs in clinical trials. For example, if only a small subset of patients benefits from a given therapy, the success of these responders will likely be “diluted” and lost in the statistics of the larger trial if the subset cannot be adequately identified and analyzed. PET/CT(MRI) with [^18^F]TFAHA may identify GBMs with high levels of HDACs class IIa expression-activity which could be treated with a combination of Tasquinimod (HDAC4 allosteric inhibitor) plus TMZ and radiation, and may potentially improve the outcome of chemo-radiation therapy and to prolong the overall survival of patients with GBM.

## Conclusions

PET/CT/(MRI) with [^18^F]TFAHA is an effective molecular imaging approach for non-invasive, quantitative, and repetitive visualization of HDACs class IIa expression-activity in glioma models in rats, as well as for monitoring the efficacy of pharmacologic inhibition by HDACs class IIa specific inhibitors. Because PET/CT/(MRI) with [^18^F]TFAHA is readily translatable into the clinic, it can aid in the selection of patients with high levels of HDACs class IIa expression-activity, who may benefit from HDACs class IIa inhibitors in combination with TMZ and radiation therapy. Furthermore, PET/CT/(MRI) with [^18^F]TFAHA could be used for monitoring the pharmacodynamics and optimization of doses of novel HDACs class IIa specific inhibitors in early phase clinical trials.

## Materials and Methods

Animal care and use procedures were carried out in accordance with protocols written under the guidelines of the National Institutes of Health Guide for the Care and Use of Laboratory Animals and approved by the Institutional Animal Care and Use Committee (IACUC) of Wayne State University. During the surgical and imaging procedures, the rats were anesthetized by inhalation of isoflurane (5% in oxygen for induction, and 2–2.5% for maintenance) and their body temperature was maintained using electronically-controlled heating pad (M2M Imaging, Cleveland, OH) set at 37 °C.

### Intracerebral Tumor Implantation

U87-MG human glioma and 9L rat gliosarcoma cells were obtained from the American Tissue Culture Collection (ATCC, Manassas, VA) and propagated in tissue culture-treated T75 flasks (Corning, Tewksbury, MA). The 9L and U87-MG cells were and cultured in DMEM or EMEM, respectively, supplemented with 10% FBS and 1% penicillin/streptomycin. For intracerebral (i.c.) injection of tumor cells, the culture media was aspirated and cells were dislodged using 0.25% trypsin (Thermo Fisher, Waltham, MA). Trypsin was inactivated using culture medium containing 10% FBS (Hyclone, Logan, UT); then, the cell suspension was centrifuged to obtain a cell pellet, which was re-suspended in cell culture medium without serum to achieve the following concentrations: U87-MG (4 × 10^5^ in 20 µL) and 9L (1 × 10^5^ in 10 µL). Cell suspensions were kept at 2–4 °C in an ice bath for no longer than 30 min. Male Sprague Dawley rats (Envigo, Indianapolis, IN, 400–500 g) were used to generate the 9L glioma model (N = 10) and athymic *rnu/rnu* rats (300–350 g, Taconic Biosciences, NY) to generate the U87-MG model (N = 9). The top of the anesthetized rat’s head was shaved, fixed in a stereotaxic frame (Kopf-Tujunga, Germany), and the skull exposed via a midline incision. A burr hole was created using a micro-drill with a 2.3 mm tip (CellPoint Scientific, Gaithersburg, MD). A short-beveled 26-ga needle attached to the 50 µL Hamilton syringe (Hamilton Company, Reno, NV), containing tumor cell suspension, was inserted into the brain −1.5 AP, −4 mm LAT, −6 mm DV relative to *bregma*. The tumor cell suspension was slowly injected into the brain parenchyma over the period of 10 minutes to ensure steady resorption of injectate by the brain and to prevent the back-flux of cells into the subarachnoid and subdural spaces. After the needle was withdrawn, the hole in the dura was closed by cauterization, the burr hole filled with bone wax (Medline, Northfield, Il), and the skin incision closed using 3–0 black silk running suture (Ethicon, Somerville, NJ). The rats were monitored post-operatively for signs of distress, weight loss, or neurological deficit, and administered fluids (i.e., saline by subcutaneous injection) or nutritional supplements, as needed.

### MR Imaging

T2-weighted MRI was obtained 14 days after intracerebral tumor implantation to verify tumor localization, size, and extent of peritumoral edema. The animals were held in position using a bite bar and a home-built receive-only surface coil 2-element phased array was placed dorsal to the head, as described elsewhere^78,79^. Images were acquired using a 7T ClinScan (Bruker, UK) preclinical MRI system operated by a Siemens console equipped with Syngo software (Siemens, Knoxville, TN). Coronal and axial T2-weighted images were obtained using the following settings: repetition time (TR) 3530 ms, echo time (TE) 38 ms, slice thickness 0.5 mm, FOV 3.2 cm × 3.2 cm, resolution 125 µm × 125 µm × 1 mm, matrix 320 × 320.

### PET/CT Imaging

The radiosynthesis and formulation of [^18^F]TFAHA for intravenous (i.v.) injection was performed as previously described^42^. Under inhalation anesthesia (as described above), the rat head fixed in a stereotactic holder made of polycarbonate plastic (Kopf-Tujunga, Germany), attached to the bed of microPET R4 scanner (Siemens, Knoxville, TN), and the head was positioned in the center of the field of view (FOV). Then, the rat was injected via the tail vein with [^18^F]TFAHA (350–500 µCi in 1 ml), as a steady injection over 1 min; PET images were obtained in a dynamic mode over 30 minutes post radiotracer administration. Thereafter, the detachable bed with the affixed animal was transferred into the Inveon SPECT/CT scanner (Siemens, Knoxville, TN) and CT images of the head were acquired using 80 kV and 500 uA current settings with exposure time of 300–350 milliseconds at each of the 360 rotational steps.

### Reconstruction, Co-registration and Analysis of Multi-Modality Images

Dynamic PET datasets were truncated into multiple 1–2 min static frames and images reconstructed using two-dimensional ordered subsets expectation maximization (2DOSEM) algorithm with four iterations and 16 subsets^42^. CT images were reconstructed using Shepp–Logan algorithm^80^ and PET/CT image fusion was accomplished using Inveon Research Workplace version 3.0 software package (Siemens, Knoxville, TN). PET/CT and T2-weighted MR images of individual rat heads were manually co-registered using skull landmarks as fiducial markers the AMIDE 1.0.4 software. Digital rat brain atlas^81^ was used for identification of specific structures of the brain and manual segmentation of T2-weighted MR images, based on stereotactic coordinates with spatial corrections for deformation and displacement of brain structures by space-occupying tumor lesions and peritumoral edema. Radioactivity concentration in tumors and specific brain structures were quantified with the AMIDE 1.0.4 software using regions of interest (ROI) analysis and expressed as Bq/g and SUV. Time-activity curves (TAC) for intracerebral tumors and other brain structures are plotted over time post radiotracer administration. Tumor-to-cortex and tumor-to-muscle ratios of radioactivity concentration, relative volumes of distribution, were determined at different time points using SUV ratios as described by us before^42^.

### PET/CT/(MRI) with [^18^F]TFAHA for monitoring the pharmacologic inhibition of HDACs class IIa expression-activity in intracerebral 9L gliomas in rats

Two days after initial [^18^F]TFAHA PET/CT/(MRI) studies (baseline), rats bearing intracerebral 9L gliomas were treated either with HDACs class IIa inhibitor MC1568 (30 mg/kg, i.p.; N = 3) or with SIRT1-specific inhibitor EX-527 (5 mg/kg, i.p.; N = 3) and 30 minutes later were injected i.v. with 250 µCi of [^18^F]TFAHA. PET/CT images were acquired in dynamic mode over the period of 60 min. To facilitate comparative analyses, PET/CT/(MRI) images acquired after MC1568 and EX-527 treatments were co-registered with pre-treatment (baseline) images. [^18^F]TFAHA SUVs before and after treatments with MC1568 or EX-527 were calculated for the same regions of interest (ROI) and compared. Time-activity curves (TACs) were generated from the dynamic PET datasets and Logan graphical analysis^82^ for reversibly accumulating radiotracers was used to calculate the apparent tissue distribution volumes (*DV*) of [^18^F]TFAHA-derived radioactivity in 9L tumors and different structures of the brain. Brain cortex was used as a reference tissue for the Logan graphical analysis^83^, because brain cortex expresses lower levels of HDACs 4 and 5 than other structures of the brain^47^ and has high blood perfusion.

### Histology and Immunohistochemistry

Following completion of imaging studies, the results of PET/CT(MRI) were validated by histological and immunohistochemical analyses. Prior to euthanasia, the animals were anesthetized with an i.p. injection of sodium pentobarbital (50 mg/kg) and transcardially perfused with 400 ml of normal saline followed by 400 ml of 4% paraformaldehyde in PBS (pH 7.4); brain was removed and placed in PBS with 4% paraformaldehyde and 30% sucrose for two days, then transferred to PBS with 30% sucrose. Thereafter, the brain was frozen on dry ice, imbedded in OCT matrix (Thermo Fischer, Waltham, MA), and serial 20 μM frozen brain sections were obtained at −20 °C in either coronal or axial planes using OTF5000 cyromicrotome (Hacker–Bright Instruments, Winnsboro, SC). Brain tissue sections were placed on high tissue-binding glass slides (Superfrost Plus; Thermo Fisher, Waltham, MA), heat-fixed at 55 °C using a slide warmer Premiere XH-2001 (C&A Scientific, Manassas, VA), and subsequently stored at −80 °C. For immunohistochemical (IHC) staining, brain sections were washed in 0.1 M PBS (pH 7.4) 3 times with 3 min intervals. Sections were progressively dehydrated using graded alcohols (25%, 50%, 75%, and 100%) with 3 × 3-minute intervals and then washed in xylene for 5 min. Antigen retrieval was achieved by heating the sections at 75 °C for 1 hour in sodium citrate buffer solution (pH 6.0). Thereafter, tissue sections were washed in PBS 3 × 3-min intervals, followed by immersion in 0.6% H_2_O_2_ in PBS for 1 hour to suppress endogenous peroxidase activity. Then, tissue sections were rinsed once again in PBS 3 × 3 min intervals and incubated overnight with one of the following rabbit polyclonal primary antibodies: HDAC4 (H-92, 1:50, Santa Cruz Biotechnology, CA), HDAC5 (H-74, 1:50, Santa Cruz Biotechnology, CA), HDAC 9 (H-45, 1:50 Santa Cruz Biotechnology, CA), acetyl-H2AK5 (#2576, 1:100, Cell Signaling, MA), acetyl-H2BK5 (#2574, 1:100, Cell Signaling, MA), acetyl-H3K9 (#9711, 1:500, Cell Signaling, MA) or acetyl-H4K8 (#2594, 1:1500, Cell Signaling, MA), HIF-1α (mgc3, 1:100, Thermo Fischer, Waltham, MA), and KLF4 (1E6, 1:100, Thermo Fisher, Waltham, MA). On the following day, the sections were washed in PBS 3 × 3-minute intervals and incubated for 1.5 hours with goat-anti-rabbit biotynilated secondary antibodies (Vectastain Elite Kit, Vector Laboratories, Burlingame, CA), then washed in PBS 3 × 3-minute intervals and incubated in avidin-peroxidase complex (Vector Laboratories, Burlingame, CA) for 1 hour. Thereafter, tissue sections were washed in PBS 3 × 3-minute intervals and incubated for 90 seconds in 0.05% 3,3-diaminobenzadine and 0.015% H_2_O_2_ (Sigma-Aldrich, St. Louis, MO) in water, then washed in tap water, counterstained with hematoxylin and eosin (H&E; Thermo Fisher, Waltham, MA) using a conventional protocol, and cover-slipped using Surgipath Micromount medium (Leica, Germany). Evaluation IHC-stained brain tissue sections and acquisition of images was performed using EVOS FL Auto microscope (Life Technologies, Carlsbad, CA).

### Statistical Analyses

Numerical and statistical analyses of data were performed using Excel 2010 (Microsoft, Redmond, WA) and Graph-Pad Prism 6 (Graph Pad Software La Jolla, CA). Student t-test for group mean and paired measurements were performed to calculate P values. α of 0.05 was used as the threshold to indicate a significant difference, and a two-tailed distribution was assumed. p < 0.05 was considered as statistically significant. Group means ± standard errors were calculated for SUVs and distribution volumes (DV) in tumor and contralateral brain structures (*hippocampus*, *n*. *accumbens*, white matter), cerebellum, and periadequctal grey for multiple time points (0, 5, 8, 10, 12, 15, 20, 25, 30 min) post-injection and compared across the cell lines using one-way ANOVA.

## Supplementary information

Supplementary Information Figures S1-S5
