# Supplementary material for: Molecular imaging HDACs class IIa expression-activity and pharmacologic inhibition in intracerebral glioma models in rats using PET/CT/(MRI) with [18F]TFAHA
Source: Sci Rep. 2019 Mar 5;9:3595. doi: 10.1038/s41598-019-40054-2 (PMC6401080; doi:10.1038/s41598-019-40054-2)
Supplement: Supplementary file 1 — Supplementary Information Figures S1-S5 [file 41598_2019_40054_MOESM1_ESM.pdf]

Molecular imaging HDACs class IIa expression-activity and pharmacologic inhibition in intracerebral glioma models in rats using PET/CT/(MR) with [^{18}F]TFAHA.

Maxwell T. Laws[1], Robin E. Bonomi[1], Swatabdi Kamal[1], David J. Gelovani[1], Jeremy Llaniguez[1], Shreya Potukutchi[1], Xin Lu[2], Thomas Mangner[2], and Juri G. Gelovani[1,3,4,5]*

1) Department of Biomedical Engineering, College of Engineering and School of Medicine, Wayne State University, Detroit, MI 48202, USA
2) Positron Emission Tomography Center, Wayne State University, Detroit, MI 48202, USA
3) Department of Oncology, Wayne State University, Detroit, MI 48202, USA
4) Department of Neurosurgery, Wayne State University, Detroit, MI 48202, USA
5) Molecular Imaging Program, Karmanos Cancer Institute, Wayne State University, Detroit, MI, 48201, USA

* Correspondence and requests for materials should be addressed to J.G.G. (email: juri.gelovani@wayne.edu)

Figure S1

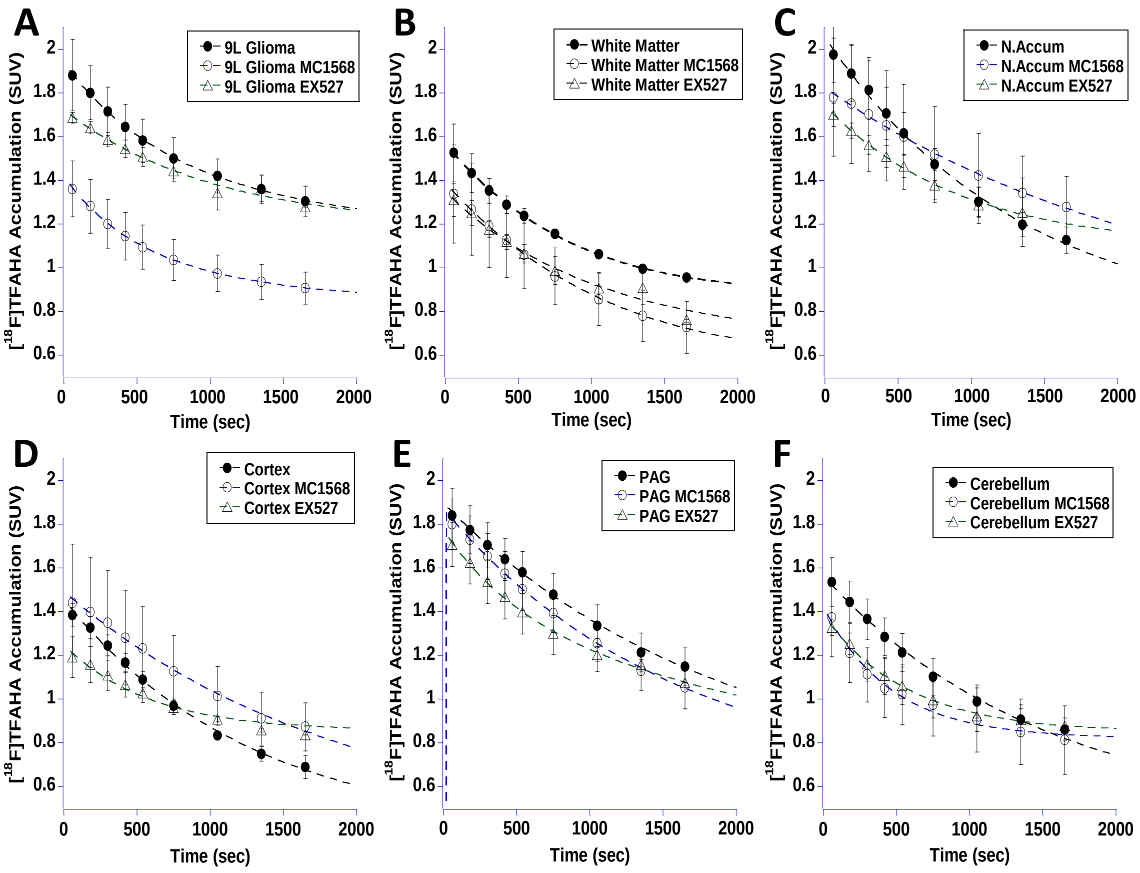

A
9L Glioma
9L Glioma MC1568
9L Glioma EX527
B
White Matter
White Matter MC1568
White Matter EX527
C
N.Accum
N.Accum MC1568
N.Accum EX527
D
Cortex
Cortex MC1568
Cortex EX527
E
PAG
PAG MC1568
PAG EX527
F
Cerebellum
Cerebellum MC1568
Cerebellum EX527
[18F]TFAHA Accumulation (SUV)
Time (sec)

Figure S1. Time-activity curves of [^{18}F]TFAHA after intravenous administration in the brain of rats bearing intracerebral 9L gliomas: **A**) 9L gliomas; **B**) white matter; **C**) *n.accumbens*; **D**) cortex; **E**) periaqueductal gray (PAG) matter; **F**) cerebellum. Data points in the time-activity curves determined under different conditions are: before treatment - closed circles; after treatment with MC1568 – open circles; after treatment with EX-527 – open triangles. Data points: mean±SEM. The magnitude of [^{18}F]TFAHA-derived radioactivity in individual regions is presented as standard uptake value (SUV). Time scale is in seconds (sec).

The magnitude of [^{18}F]TFAHA time-activity curves (TACs) was statistically significantly decreased ($p<0.005$) in 9L gliomas after pre-treatment of animals with MC1568, whereas no significant differences in the magnitude and profiles of [^{18}F]TFAHA TACs were observed after pre-treatment with EX-527 (**Fig. S1**). Also, no statistically significant decreases in [^{18}F]TFAHA TACs were observed in the contralateral normal brain structures known to overexpress HDACs class IIa (i.e., n.accumbens, PAG, cerebellum).

Figure S2

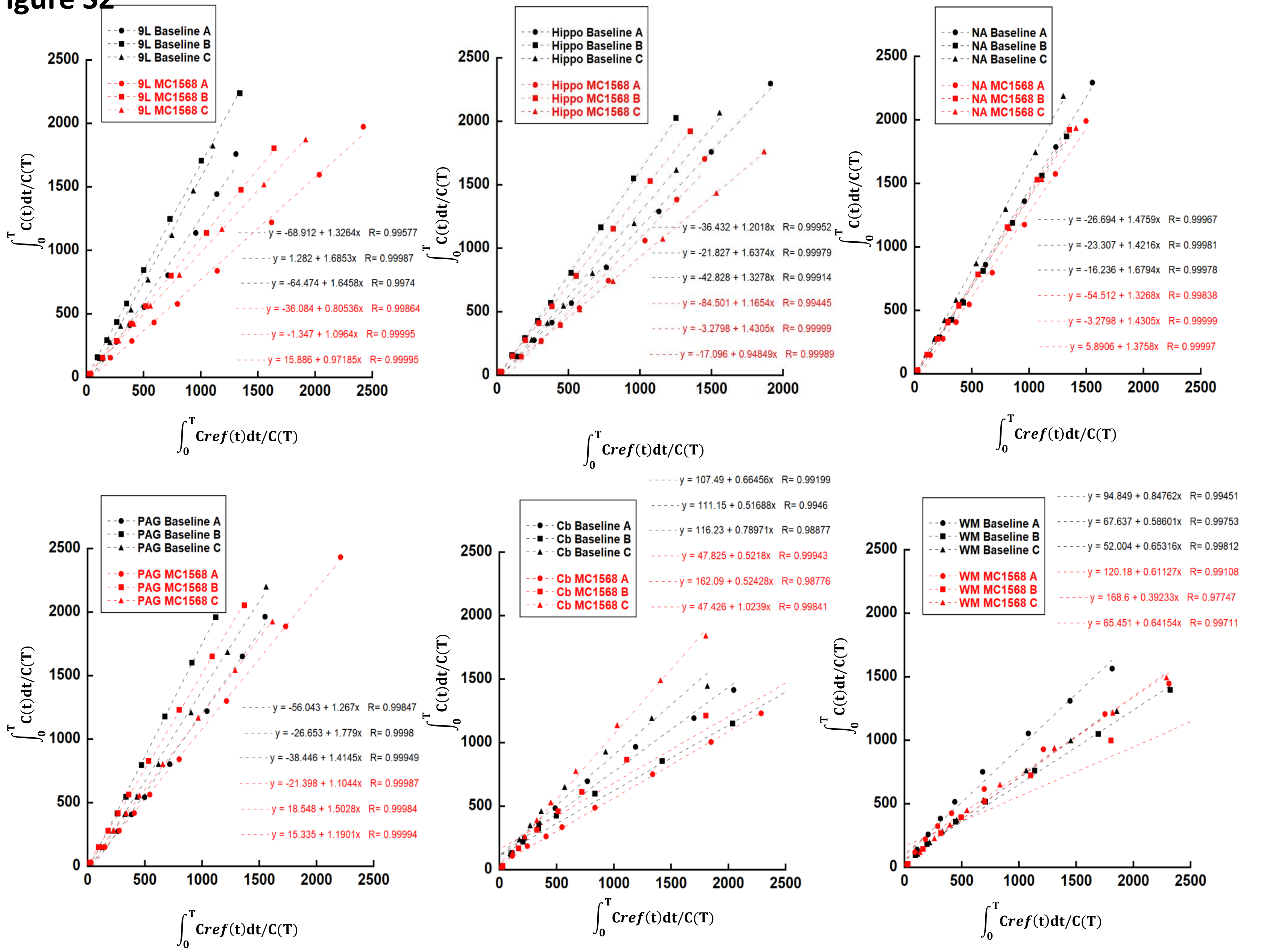
9L Baseline A
9L Baseline B
9L Baseline C
9L MC1568 A
9L MC1568 B
9L MC1568 C
y = -68.912 + 1.3264x R= 0.99577
y = 1.282 + 1.6853x R= 0.99987
y = -64.474 + 1.6458x R= 0.9974
y = -36.084 + 0.80536x R= 0.99864
y = -1.347 + 1.0964x R= 0.99995
y = 15.886 + 0.97185x R= 0.99995
$\int_0^T C(t)dt/C(T)$
$\int_0^T Cref(t)dt/C(T)$
Hippo Baseline A
Hippo Baseline B
Hippo Baseline C
Hippo MC1568 A
Hippo MC1568 B
Hippo MC1568 C
y = -36.432 + 1.2018x R= 0.99952
y = -21.827 + 1.6374x R= 0.99979
y = -42.828 + 1.3278x R= 0.99914
y = -84.501 + 1.1654x R= 0.99445
y = -3.2798 + 1.4305x R= 0.99999
y = -17.096 + 0.94849x R= 0.99989
$\int_0^T C(t)dt/C(T)$
$\int_0^T Cref(t)dt/C(T)$
NA Baseline A
NA Baseline B
NA Baseline C
NA MC1568 A
NA MC1568 B
NA MC1568 C
y = -26.694 + 1.4759x R= 0.99967
y = -23.307 + 1.4216x R= 0.99981
y = -16.236 + 1.6794x R= 0.99978
y = -54.512 + 1.3268x R= 0.99838
y = -3.2798 + 1.4305x R= 0.99999
y = 5.8906 + 1.3758x R= 0.99997
$\int_0^T C(t)dt/C(T)$
$\int_0^T Cref(t)dt/C(T)$
PAG Baseline A
PAG Baseline B
PAG Baseline C
PAG MC1568 A
PAG MC1568 B
PAG MC1568 C
y = -56.043 + 1.267x R= 0.99847
y = -26.653 + 1.779x R= 0.9998
y = -38.446 + 1.4145x R= 0.99949
y = -21.398 + 1.1044x R= 0.99987
y = 18.548 + 1.5028x R= 0.99984
y = 15.335 + 1.1901x R= 0.99994
$\int_0^T C(t)dt/C(T)$
$\int_0^T Cref(t)dt/C(T)$
y = 107.49 + 0.66456x R= 0.99199
y = 111.15 + 0.51688x R= 0.9946
y = 116.23 + 0.78971x R= 0.98877
y = 47.825 + 0.5218x R= 0.99943
y = 162.09 + 0.52428x R= 0.98776
y = 47.426 + 1.0239x R= 0.99841
Cb Baseline A
Cb Baseline B
Cb Baseline C
Cb MC1568 A
Cb MC1568 B
Cb MC1568 C
$\int_0^T C(t)dt/C(T)$
$\int_0^T Cref(t)dt/C(T)$
y = 94.849 + 0.84762x R= 0.99451
y = 67.637 + 0.58601x R= 0.99753
y = 52.004 + 0.65316x R= 0.99812
y = 120.18 + 0.61127x R= 0.99108
y = 168.6 + 0.39233x R= 0.97747
y = 65.451 + 0.64154x R= 0.99711
WM Baseline A
WM Baseline B
WM Baseline C
WM MC1568 A
WM MC1568 B
WM MC1568 C
$\int_0^T C(t)dt/C(T)$
$\int_0^T Cref(t)dt/C(T)$

Figure S2. Logan graphical analyses of transient retention of [^{18}F]TFAHA-derived radioactivity in 9L tumors and different brain structures in the same animals (N=3) at baseline (black) and after therapy with MC1568 (red); Data points for individual animals are shown in different shapes (circle, square, triangle). Panels: 9L (9L glioma), Hippo (contralateral *hippocampus*), NA (contralateral *n.accumbens*), PAG (periaqueductal grey), Cb (cerebellum), WM (contralateral white matter). Linear regression equations for individual animal data points are listed in the same sequence as the data legends. Linear regression coefficients represent DVs of [^{18}F]TFAHA-derived radioactivity in 9L tumors and various structures of the brain.

Figure S3

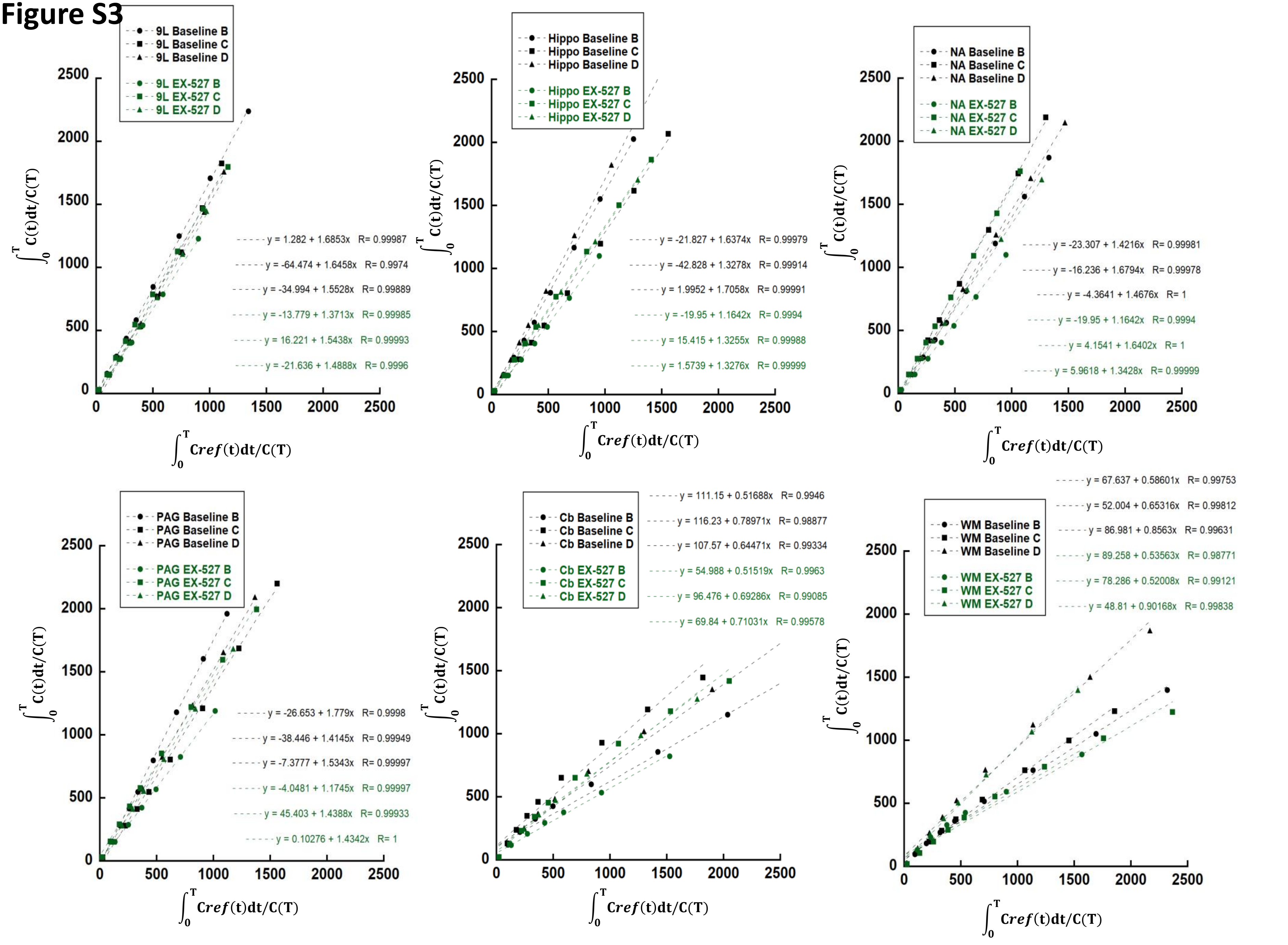
9L Baseline B
9L Baseline C
9L Baseline D
9L EX-527 B
9L EX-527 C
9L EX-527 D
y = 1.282 + 1.6853x R= 0.99987
y = -64.474 + 1.6458x R= 0.9974
y = -34.994 + 1.5528x R= 0.99889
y = -13.779 + 1.3713x R= 0.99985
y = 16.221 + 1.5438x R= 0.99993
y = -21.636 + 1.4888x R= 0.9996
Hippo Baseline B
Hippo Baseline C
Hippo Baseline D
Hippo EX-527 B
Hippo EX-527 C
Hippo EX-527 D
y = -21.827 + 1.6374x R= 0.99979
y = -42.828 + 1.3278x R= 0.99914
y = 1.9952 + 1.7058x R= 0.99991
y = -19.95 + 1.1642x R= 0.9994
y = 15.415 + 1.3255x R= 0.99988
y = 1.5739 + 1.3276x R= 0.99999
NA Baseline B
NA Baseline C
NA Baseline D
NA EX-527 B
NA EX-527 C
NA EX-527 D
y = -23.307 + 1.4216x R= 0.99981
y = -16.236 + 1.6794x R= 0.99978
y = -4.3641 + 1.4676x R= 1
y = -19.95 + 1.1642x R= 0.9994
y = 4.1541 + 1.6402x R= 1
y = 5.9618 + 1.3428x R= 0.99999
PAG Baseline B
PAG Baseline C
PAG Baseline D
PAG EX-527 B
PAG EX-527 C
PAG EX-527 D
y = -26.653 + 1.779x R= 0.9998
y = -38.446 + 1.4145x R= 0.99949
y = -7.3777 + 1.5343x R= 0.99997
y = -4.0481 + 1.1745x R= 0.99997
y = 45.403 + 1.4388x R= 0.99933
y = 0.10276 + 1.4342x R= 1
Cb Baseline B
Cb Baseline C
Cb Baseline D
Cb EX-527 B
Cb EX-527 C
Cb EX-527 D
y = 111.15 + 0.51688x R= 0.9946
y = 116.23 + 0.78971x R= 0.98877
y = 107.57 + 0.64471x R= 0.99334
y = 54.988 + 0.51519x R= 0.9963
y = 96.476 + 0.69286x R= 0.99085
y = 69.84 + 0.71031x R= 0.99578
WM Baseline B
WM Baseline C
WM Baseline D
WM EX-527 B
WM EX-527 C
WM EX-527 D
y = 67.637 + 0.58601x R= 0.99753
y = 52.004 + 0.65316x R= 0.99812
y = 86.981 + 0.8563x R= 0.99631
y = 89.258 + 0.53563x R= 0.98771
y = 78.286 + 0.52008x R= 0.99121
y = 48.81 + 0.90168x R= 0.99838
∫0T C(t)dt/C(T)
∫0T Cref(t)dt/C(T)

Figure S3. Logan graphical analyses of transient retention of [^{18}F]TFAHA-derived radioactivity in 9L tumors and different brain structures in the same animals (N=3) at baseline (black) and after therapy with EX-527 (green); Data points for individual animals are shown in different shapes (circle, square, triangle). Panels: 9L (9L glioma), Hippo (contralateral *hippocampus*), NA (contralateral *n.accumbens*), PAG (periaqueductal grey), Cb (cerebellum), WM (contralateral white matter). Linear regression equations for individual animal data points are listed in the same sequence as the data legends. Linear regression coefficients represent DVs of [^{18}F]TFAHA-derived radioactivity in 9L tumors and various structures of the brain.

Figure S4

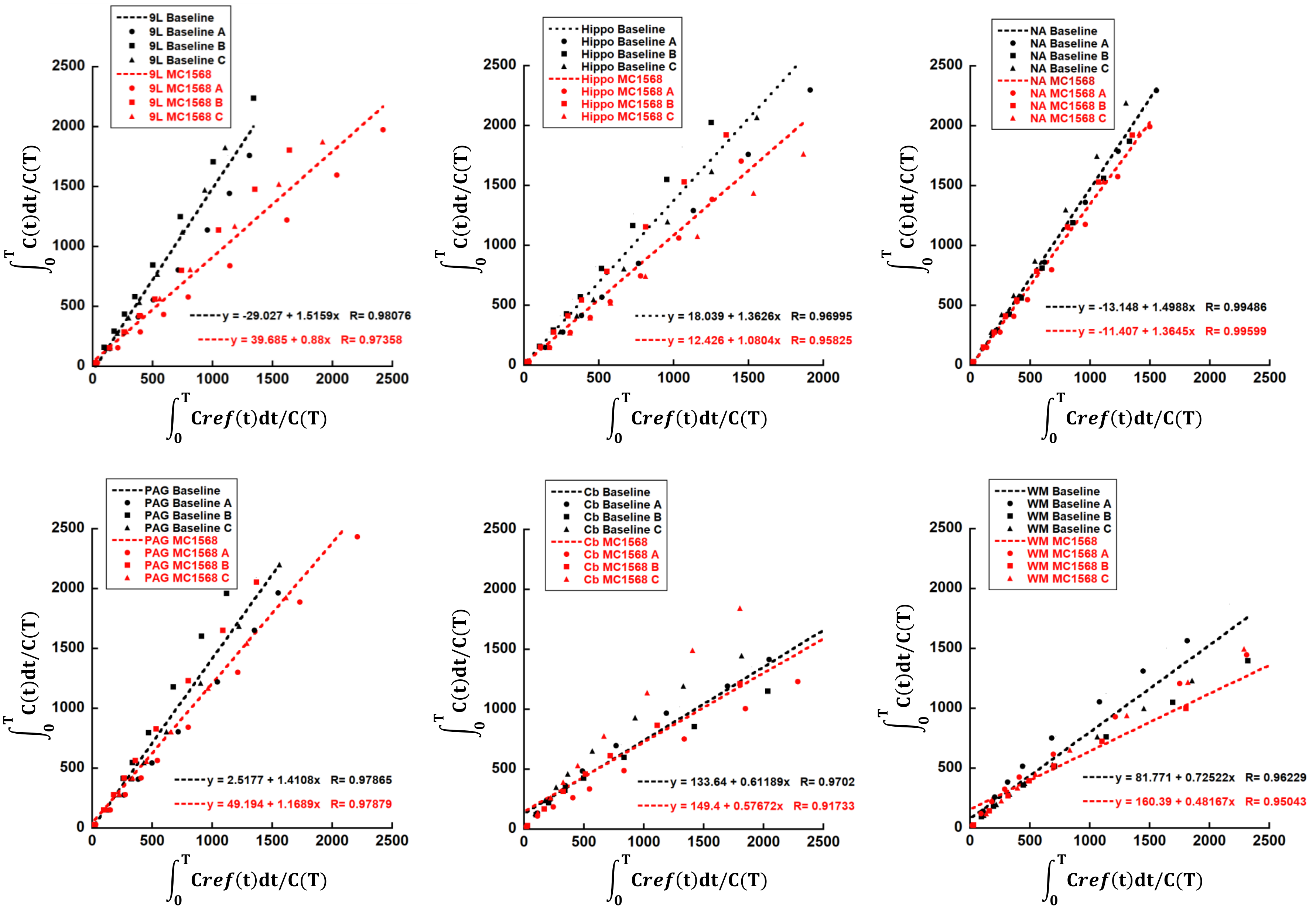

9L Baseline
9L Baseline A
9L Baseline B
9L Baseline C
9L MC1568
9L MC1568 A
9L MC1568 B
9L MC1568 C
y = -29.027 + 1.5159x R= 0.98076
y = 39.685 + 0.88x R= 0.97358
Hippo Baseline
Hippo Baseline A
Hippo Baseline B
Hippo Baseline C
Hippo MC1568
Hippo MC1568 A
Hippo MC1568 B
Hippo MC1568 C
y = 18.039 + 1.3626x R= 0.96995
y = 12.426 + 1.0804x R= 0.95825
NA Baseline
NA Baseline A
NA Baseline B
NA Baseline C
NA MC1568
NA MC1568 A
NA MC1568 B
NA MC1568 C
y = -13.148 + 1.4988x R= 0.99486
y = -11.407 + 1.3645x R= 0.99599
PAG Baseline
PAG Baseline A
PAG Baseline B
PAG Baseline C
PAG MC1568
PAG MC1568 A
PAG MC1568 B
PAG MC1568 C
y = 2.5177 + 1.4108x R= 0.97865
y = 49.194 + 1.1689x R= 0.97879
Cb Baseline
Cb Baseline A
Cb Baseline B
Cb Baseline C
Cb MC1568
Cb MC1568 A
Cb MC1568 B
Cb MC1568 C
y = 133.64 + 0.61189x R= 0.9702
y = 149.4 + 0.57672x R= 0.91733
WM Baseline
WM Baseline A
WM Baseline B
WM Baseline C
WM MC1568
WM MC1568 A
WM MC1568 B
WM MC1568 C
y = 81.771 + 0.72522x R= 0.96229
y = 160.39 + 0.48167x R= 0.95043
$\int_0^T C(t)dt/C(T)$
$\int_0^T Cref(t)dt/C(T)$

Figure S4. Logan graphical analyses of transient retention of [^{18}F]TFAHA-derived radioactivity in 9L tumors and different brain structures of the same group animals (N=3) at baseline (black) and after therapy with MC1568 (red); Data points for individual animals are shown in different shapes (circle, square, triangle). Panels: 9L (9L glioma), Hippo (contralateral *hippocampus*), NA (contralateral *n.accumbens*), PAG (periaqueductal grey), Cb (cerebellum), WM (contralateral white matter). Linear regression lines and equations for the group data points are shown for baseline (black) and after treatment with MC1568 (red). Linear regression coefficients represent DVs of [^{18}F]TFAHA-derived radioactivity in 9L tumors and various structures of the brain.

Figure S5

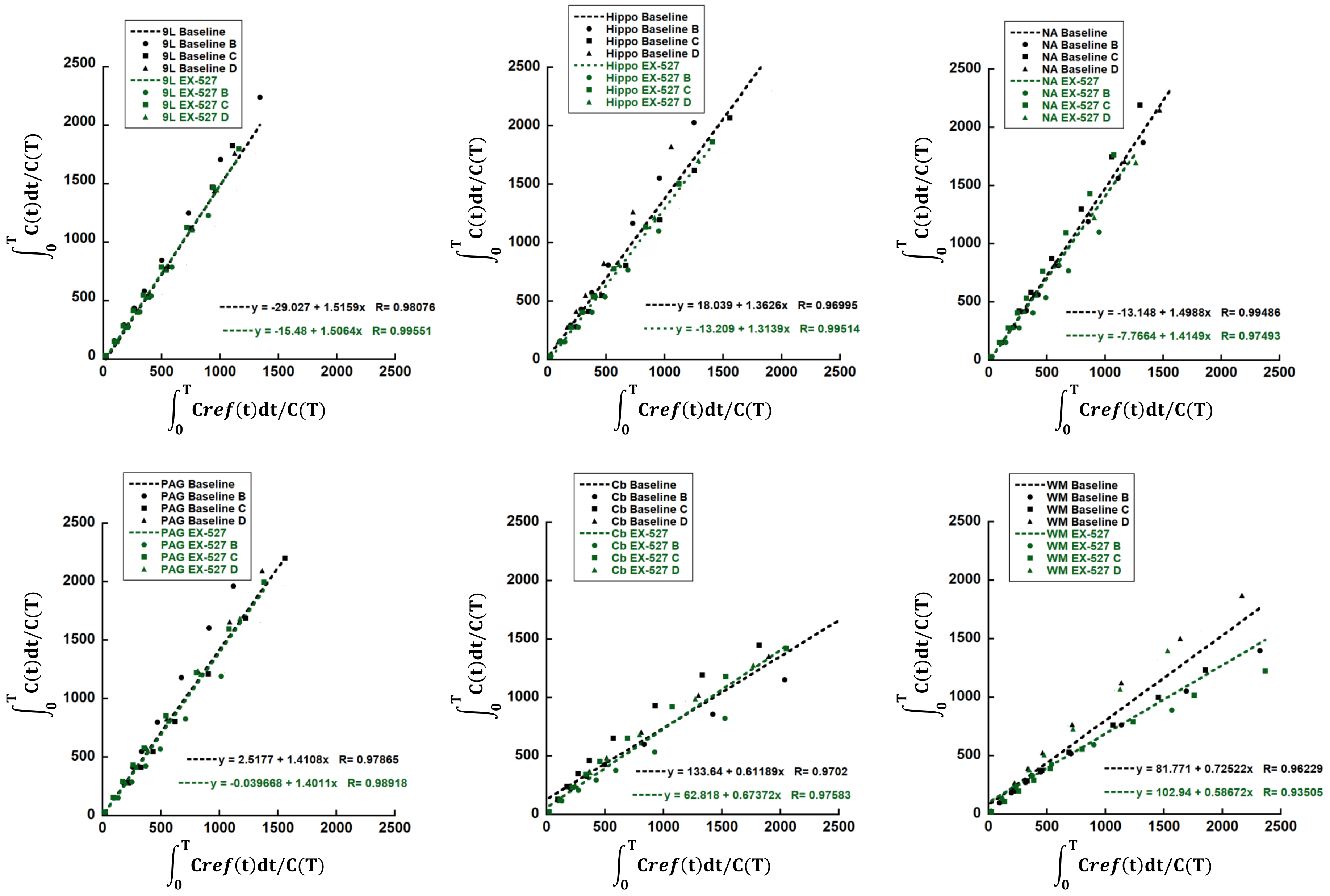
9L Baseline
9L Baseline B
9L Baseline C
9L Baseline D
9L EX-527
9L EX-527 B
9L EX-527 C
9L EX-527 D
y = -29.027 + 1.5159x R= 0.98076
y = -15.48 + 1.5064x R= 0.99551
Hippo Baseline
Hippo Baseline B
Hippo Baseline C
Hippo Baseline D
Hippo EX-527
Hippo EX-527 B
Hippo EX-527 C
Hippo EX-527 D
y = 18.039 + 1.3626x R= 0.96995
y = -13.209 + 1.3139x R= 0.99514
NA Baseline
NA Baseline B
NA Baseline C
NA Baseline D
NA EX-527
NA EX-527 B
NA EX-527 C
NA EX-527 D
y = -13.148 + 1.4988x R= 0.99486
y = -7.7664 + 1.4149x R= 0.97493
PAG Baseline
PAG Baseline B
PAG Baseline C
PAG Baseline D
PAG EX-527
PAG EX-527 B
PAG EX-527 C
PAG EX-527 D
y = 2.5177 + 1.4108x R= 0.97865
y = -0.039668 + 1.4011x R= 0.98918
Cb Baseline
Cb Baseline B
Cb Baseline C
Cb Baseline D
Cb EX-527
Cb EX-527 B
Cb EX-527 C
Cb EX-527 D
y = 133.64 + 0.61189x R= 0.9702
y = 62.818 + 0.67372x R= 0.97583
WM Baseline
WM Baseline B
WM Baseline C
WM Baseline D
WM EX-527
WM EX-527 B
WM EX-527 C
WM EX-527 D
y = 81.771 + 0.72522x R= 0.96229
y = 102.94 + 0.58672x R= 0.93505
$\int_0^T C(t)dt/C(T)$
$\int_0^T Cref(t)dt/C(T)$
0
500
1000
1500
2000
2500

Figure S5. Logan graphical analyses of transient retention of [^{18}F]TFAHA-derived radioactivity in 9L tumors and different brain structures of the same group animals (N=3) at baseline (black) and after therapy with EX-527 (green); Data points for individual animals are shown in different shapes (circle, square, triangle). Panels: 9L (9L glioma), Hippo (contralateral *hippocampus*), NA (contralateral *n.accumbens*), PAG (periaqueductal grey), Cb (cerebellum), WM (contralateral white matter). Linear regression lines and equations for the group data points are shown for baseline (black) and after treatment with EX-527 (green). Linear regression coefficients represent DVs of [^{18}F]TFAHA-derived radioactivity in 9L tumors and various structures of the brain.
